# Bone Cement Solidifiliation Influence the Limb Alignment and Gap Balance during TKA

**DOI:** 10.1155/2015/109402

**Published:** 2015-01-22

**Authors:** Dongquan Shi, Xingquan Xu, Anyun Guo, Jin Dai, Zhihong Xu, Dongyang Chen, Qing Jiang

**Affiliations:** ^1^The Center of Diagnosis and Treatment for Joint Disease, Drum Tower Hospital Affiliated to Medical School of Nanjing University, Zhongshan Road 321, Nanjing, Jiangsu 210008, China; ^2^Laboratory for Bone and Joint Diseases, Model Animal Research Center, Nanjing University, Nanjing, Jiangsu 210008, China

## Abstract

*Introduction*. Mechanical alignment deviation after total knee arthroplasty is a major reason for early loosening of the prosthesis. Achieving optimum cement penetration during fixation of the femoral and tibial component is an essential step in performing a successful total knee arthroplasty. Bone cement is used to solidify the bone and prosthesis. Thickness imbalance of bone cement leads to the deviation of mechanical alignment. To estimate the influence of bone cement, a retrospective study was conducted. *Materials and Methods*. A total of 36 subjects were studied. All the TKA were performed following the standard surgical protocol for navigated surgery by medial approach with general anaesthesia. Prostheses were fixed by bone cement. *Results*. We compared the mechanical axis, flexion/extension, and gap balance before and after cementation. All the factors were different compared with those before and after cementation. Internal rotation was reached with statistical significance (*P* = 0.03). *Conclusion*. Bone cement can influence the mechanical axis, flexion/extension, and gap balance. It also can prompt us to make a change when poor knee kinematics were detected before cementation.

## 1. Introduction

Mechanical alignment deviation after total knee arthroplasty (TKA) may lead to early symptom and loosening of the prosthesis. Mechanical axis is defined by the center of the femoral head and the center of talus. The overall postoperative limb alignment should be corrected to within 0°  ± 3° of the mechanical axis [1]. To restore the correct mechanical alignment is the most important issue during TKA. Bone resection and soft tissue release are two major tools for correcting the mechanical alignment after TKA [2].

Achieving optimum cement penetration during fixation of the femoral and tibial component is an essential step in performing a successful TKA. Cement penetration uniformly with 3–5 mm below the prosthesis can improve the static strength of the implant-cement-bone construct and to ensure the long-term mechanical fixation of the implant by preventing the infiltration of wear particles, thereby avoiding peripheral osteolysis and associated component loosening [3–6]. Conversely, nonuniformly cement penetration can cause mechanical alignment deviation. For the severe osteoporosis patients with rheumatoid arthritis, bone cement pressurization might lead to collapse fracture and poor mechanical axis would happen. Previous reports showed some techniques were used to enhance the mechanical properties of the implant-cement-bone construct by improve the quality of the bone surface or cement and pressurization of the cement-bone interface [7–10]. Mechanical axis deviation and gap imbalance still occurred and finally led to aseptic loose of the prosthesis.

Bone cement is used to solidify the bone and prosthesis. Thickness imbalance of bone cement leads to the deviation of mechanical alignment. To estimate the influence of bone cement, a retrospective study was conducted. Changes were detected when the bone cement was pressurized.

## 2. Materials and Methods

### 2.1. Patients

A total of 36 subjects enrolled at the Center of Diagnosis and Treatment for Joint Disease, Drum Tower Hospital, affiliated to the Medical School of Nanjing University, were studied. All the patients received Genesis II prosthesis (Smith & Nephew). And surgeries were performed by the same surgeon (JQ). The study was approved by the ethical committee of the participating institutions, and informed consent was obtained from all subjects.

### 2.2. Surgical Technique and Measurements

All the TKA were performed following the standard surgical protocol for navigated surgery by medial approach with general anaesthesia. The bone was resected according to the instruction of navigation system. All patellae were resurfaced. For all the patients, the femur was prepared before the tibia. Soft tissue balancing was accomplished first by spacer blocks after completion of the bony resection.

Prostheses were fixed by bone cement. Two ways were used to compact the prosthesis and bone: (1) beating by the compactor; (2) extruding with thicker tibia insert. The mechanical axis, rotation of the limb, and gap balance were displayed by the navigation system and recorded. Weight bearing X-ray was taken after the operation.

## 3. Data Analysis and Statistics

Analyses were carried out to determine which way for compacting the bone cement between prosthesis and bone can reduce the influence of mechanical and rotation alignment during TKA. Differences before and after operation were tested with the Mann-Whitney test to determine whether bone cement influenced the mechanical axis and gap balance during TKA. *P* values of ≤0.05 were considered statistically significant.

## 4. Results

The demographic data and characteristics were shown in the Table 1.

38 patients (32 females and 6 males) were involved in the study. The initial low limb extremity mechanical alignment for varus knees was 7.74 ± 4.51 degrees and valgus knee was 6.67 ± 5.46 degrees. The initial knee flexion was 12.5 ± 8.8 degrees and extension was 6.90 ± 6.76 degrees. Distal femurs were resected for 9.98 ± 1.49 mm. Anterior femurs were resected for 1.46 ± 1.97 mm, and tibias were resected for 8.13 ± 2.08 mm (Table 2).

All the patients were divided into 2 groups as referred in the methods section. Before the cement was solidified, the low limb mechanical alignment with varus malalignment was 2.85 ± 2.63 degrees and 0.92 ± 0.28 degrees for valgus malalignment; the knee flexions were 5 ± 3.29 degrees and extension was 3.50 ± 3.64 degrees; for the AP rotation, the external rotation was 6 ± 3.97, and internal rotation was 3.06 ± 2.95. Lateral gap was 12.9 ± 2.26 mm and medial gap was 11.9 ± 1.69 mm (Table 3). The mechanical axis and gap balance were changed after cementation. For the AP direction, internal rotation was significant changed (*P* = 0.03). Although no statistically difference was detected, certain changes of mechanical axis and gap balance were found before and after cementation. Better mechanical axis trend was shown in patients with valgus deformity (*P* = 0.62 and 0.13, resp.). Lateral gap was broadened, while there was no difference of medial gap (Table 3).

## 5. Discussion

The most important finding of the study was that compacting bone cement between the prosthesis and bone was able to influence soft tissue balance and restoration of limb alignment. It is important to master the skill of bone cement pressurization.

Soft tissue balance is essential for optimal knee kinematics. Balanced soft tissue can make a suitable range of motion. Tight arthroplasty will increase the likelihood of flexion deformity and reduce the range of motion, while loose one can lead to pain, dysfunction, hyperextension, and accelerated wear [11]. In the clinical work, some surgeons like to compact the prosthesis to the bone by compactor. It was difficult to keep the identical power during the compaction and make bone cement well-distributed. It can be helpful for maintaining the better mechanical axis alignment. In our study, based on the navigated computer, we compared the influence by bone cement. Similar medial gap was detected during the cementation and lateral gap was broadened. Ritter et al. demonstrated that patients with postoperative flexion contracture more than 5° or hyperextension more than 10° were at increased risk of more pain, poor function, and Knee Society scores [12].

A little change was detected for mechanical axis after cementation. Mechanical axis with 3° valgus is an ideal degree. Better mechanical axis was rectified during cementation in our study. It can prompt us to adjust the mechanical axis by bone cement when unsatisfied one was detected before cementation. No flexion change was detected. The extension degree was larger and the good outcome will be detected. AP internal rotation was significantly changed. It leads to subtle impingement between femoral and tibia prosthesis detected when flexing the knee during surgery. It will accelerate the abrasion of the tibia insert. However, no clinical symptom was complained after surgery. No external rotation change was detected.

Bone cement can influence the mechanical axis, flexion/extension, and gap balance. It also can prompt us to make a change when poor knee kinematics were detected before cementation.

## Figures and Tables

**Table 1:** Demographic and clinical characteristics of 38 patients who underwent total knee arthroplasty.

Characteristic	Value
Patients, number	38
Knees, number	38
Age, yr	62 ± 12.5 year
Sex, % female	84.20%
Height	1.68 ± 0.04 m
Weight	59.3 ± 16.5 kg
HSS	42 ± 2.1
VAS	8 ± 0.7
Initial valgus (6)	6.67 ± 5.46
Initial varus (32)	7.74 ± 4.51
Initial flexion (33)	12.5 ± 8.8
Initial extension (5)	6.90 ± 6.76

**Table 2:** Surgical related factors during TKA.

Factors	Value
Femoral size	2–6
Tibial size	2–6
Insert thicker	9/11 mm
Operating time	107 ± 26 min
Tourniquet time	22 ± 7.1 min
Anteriorfemoral resection	1.65 ± 1.74 mm
Distal femoral resection	10.04 ± 1.44 mm
Tibial resection	8.16 ± 2.47 mm

**Table 3:** Mechanical axis and gap difference of before and after cementation.

Factors	Before operation	After operation	*P* value
MA			
Varus	2.85 ± 2.63	2.38 ± 2.14	0.62
Valgus	0.92 ± 0.28	1.30 ± 0.27	0.13
ROM			
Flexion	5 ± 3.29	5.65 ± 3.00	0.99
Extension	3.50 ± 3.64	3.94 ± 2.40	0.61
AP rotation			
External	6 ± 3.97	5.46 ± 3.85	0.49
Internal	3.06 ± 2.95	12 ± 8.97	**0.03**
Gap			
Lateral	12.9 ± 2.26	13.11 ± 3.09	0.29
Medial	11.9 ± 1.69	11.86 ± 2.29	0.58
